# Sanitary Regulations and Prevention of Tuberculosis

**Published:** 1896-10

**Authors:** Katharine R. Collins

**Affiliations:** Atlanta, Ga.


### SANITARY REGULATIONS AND PREVENTION OF TUBERCULOSIS.

By KATHARINE R. COLLINS, M.D.,

ATLANTA, GA.

The demonstration, beyond any reasonable doubt, that tuberculosis is of microbic origin and its classification as an infectious disease, together with the knowledge of how infection is brought about, has placed it on the list of preventable diseases; but calling it a preventable disease does not mean that it can, under the existing circumstances, be completely stamped out, but it does mean that constant effort toward the one end will diminish the death-rate, which is higher than that from any other one disease.

The Imperial Health Officer of Berlin claims that he has found one out of every third person, between the ages of 15 and 60 years, examined by him, suffering from some form of this malady. The United States claims one-seventh of the total number of deaths from it, and Michigan alone, loses fifty thousand yearly out of a population of something over one million people.

We have shown ourselves able to prevent cholera from entering this country; we have reduced the dangers of contagion from smallpox to a minimum. So why not limit the spread of tuberculosis, which is just as disastrous as either of the above in its results?

In this matter the medical profession cannot proceed alone, but good and effective legislation is necessary to control this disease, and here lie the greatest difficulties.

The medical profession, because it cannot always make plain its facts to a mind unprepared, is too often accounted a profession of theories, and more especially is this true where these theories call for a radical change in the old order of things. People do so dislike being disturbed, and unless facts can be demonstrated with spectacular effect they will not realize the necessity for action. So the only way to reach them is by force.

It is to the law that the medical profession must look for aid in this matter. So efforts should be made to instruct the law—a difficult task I will admit—but easier than the education of the masses, which will not be accomplished for many years. When the smallpox, diphtheria, and scarlet fever epidemics made such frightful ravages among us, the profession did not wait to educate the laity, but appealed to the law. People are just as ignorant, and would be just as careless to-day in these epidemics, were it not for the sharp eyes of the board of health; and every board knows that were it not invested with authority by the law, it would not be permitted to flag or disinfect houses where any one of these diseases existed.

Now it is only in degree that any difference exists between the sanitary treatment of the very actively infectious diseases and tuberculosis, excepting, perhaps, that the latter we have, like the poor, always with us. Some of the most important rules to be followed in fighting this disease cannot be carried out until we can get the co-operation of the law, but until that time we must, as individuals, enforce as many of them as practicable.

One of the first things to accomplish is; Prompt and complete destruction of effete material containing this germ.

2. To secure a supply of pure water, food, and milk for the people.

3. Report of all cases to the board of health.

4. Isolation of the patient during the stage when infection is liable. Thorough disinfection after death of room occupied by patient.

5. Regulations governing the building of houses for renting purposes.

6. Better laws regarding marriage.

7. Disposal of emigrants, suffering from this disease, upon landing in this country.

First. The destruction of effete material, etc.

Since the dried sputa from patients with pulmonary tuberculosis blown about by the wind is the most common method of infection, the pollution of public highways, buildings, conveyances, etc., by the promiscuous deposit of sputum should be made an offense punishable by law. The city should provide cuspidors, placed at proper intervals, containing an antiseptic solution; these should be cleaned twice a day and the contents destroyed.

Handkerchiefs used by phthisical patients should be burned; all clothing that cannot be washed should be frequently disinfected. All linen, as soon as discarded, should be placed in water containing bichloride of mercury or carbolic acid, preferably the former, and should not be put in with the family laundry, but should be washed separately. The room occupied by the patient should be disinfected by the health officer at least once in two months during the illness and thoroughly after death.

Next we have the supply of pure food, milk, and water. To secure this each State should have a fully equipped hygienic laboratory. The head of this laboratory should have charge of the examination of applicants for the positions of inspectors, said applicants being recommended by him to the appropriate civil body for appointment. These inspectors should take their instructions from the laboratory authorities, to whom they must make reports from time to time, and must send such samples of food, water, and milk as are deemed necessary, for both chemical and bacteriological examination.

All cattle should be tested for tuberculosis. All cattle-sheds and buildings on the premises, used as dairies or slaughter-houses, should be inspected.

The utensils, the methods of cleansing them, the means of conveying the milk or meat to the depot of distribution, and also, the condition of this depot to be looked into.

The source of the water supply, with its methods of filtration, and reservoirs, the ice factories, manufactories of canned goods, and the vegetable market should all come under the closest inspection.

Persons desiring to go into such businesses should make contracts with the city to furnish a pure article, and where fraud or carelessness is detected the municipal government should immediately take it up and investigate, and the proper penalty be exacted.

We come now to the duties of the board of health. This board should require every case of tuberculosis to be reported as soon as diagnosed. This refers, of course, to those cases that are sufficiently advanced for infection to take place. The board should then see that the patient is placed under the proper restrictions at home, or sent to the hospital, which should be provided for such cases. It would be well for the board to have printed leaves, setting forth explicitly all precautions to be observed by the patient and attendants. I have already mentioned the disinfecting of the room occupied by a phthisical patient during life and after death.

Fourth. Isolation of the patient.

On account of the chronicity of the disease this cannot be accomplished in the strictest sense of the word, nor would it be right to cut such a patient off from all communication with his own people, but by proper care much can be done to lessen the danger to others. The patient should occupy a room alone; this should be insisted upon.

This room should contain as little furniture as possible, and it should be very simple in kind. All hangings and rugs should be of such material as can be washed or destroyed. The floor should be bare, the cracks filled, then painted and varnished. The walls should also be painted and varnished, and where practicable should be so ceiled as to have no acute angles. As to the bed, blankets and not comforts, should be used, the mattress and pillows should have an extra covering of heavy sheeting, which can be easily changed, and washed. All vessels, utensils, and dishes used by the patient should be cleaned and disinfected apart from those used about the house. Persons spending much time in this room should always wear clothing that can be washed and this should be changed when mingling with the family.

The patient may go among a few people for short periods of time; he should always, on such occasions, put on clothing that has been disinfected. He must avoid crowds and should not be allowed in schoolrooms and similar places; he should not be employed in dairies, slaughter-houses, etc., nor where many employees work together.

The day may not be far off when all cities will follow the lead of Berlin and New York in regulating the building of houses for renting purposes. These two cities now require for each apartment a certain amount of light and air; they prohibit the use of basements for living-rooms, and limit the number of persons that may occupy one apartment. Also, in time, parties may be required to show a clean bill of health before procuring a marriage license. Only recently, parties afflicted with epilepsy were refused license to marry in some small city in Illinois.

In regard to immigration. Should we allow parties suffering from tuberculosis to land in this country? I think not, unless they possess the means necessary to maintain themselves in a hospital designed for the treatment of such cases. Since it is so necessary for persons having tuberculosis to be sent to a more favorable climate, railroads should provide some means of transportation for them that will prevent the exposure of other passengers.

With good sanitary laws we diminish the danger of infection, so it becomes an easy matter to cope with the inherited tendency to this disease. Here it is necessary to begin as early in the life of the patient as possible. Good, nutritious food, plenty of fresh air, exercises which tend to develop the chest, increasing the resistance to cold by cold salt sponge baths, oil rubs and the wearing of proper clothing, careful treatment during and immediately following those diseases that predispose the patient to lung trouble, and removal to a favorable climate where practicable. If these precautions are persistently and intelligently followed, those persons possessing the so-called strumous diathesis need have no fear of contracting tuberculosis.

Before closing I should like to add that I am, I think, fully aware of the many obstacles in the way of carrying out the above views, but I also firmly believe that until these measures can be accomplished we will not be able to do much toward limiting the spread of tuberculosis. So even though it is only a step at a time that we can advance, let us not waste any time in taking that step.

---

### REMOVAL OF BLOOD STAINS.

According to Dr. Blenkiser, in the *Scalpel*, surgical instruments, sponges, the hands of the operator, and other blood-stained articles, may be readily cleansed by washing them in a tepid solution of tartaric acid, and then rinsing in water without soap.—*New York Medical Times.*

